# Early Clinical Experience and Mentoring of Young Dental Students—A Qualitative Study

**DOI:** 10.3390/dj9080091

**Published:** 2021-08-06

**Authors:** Rod Moore, Simone Molsing, Nicola Meyer, Matilde Schepler

**Affiliations:** Institute of Dentistry and Oral Health Sciences, Aarhus University, 8000 Aarhus C, Denmark; simonemolsing@hotmail.com (S.M.); nicola_meyer@hotmail.com (N.M.); matildeschepler@hotmail.com (M.S.)

**Keywords:** clinical education, early clinical experience, dental students, motivation, stress perceptions, self-determination theory, self-efficacy, social learning theory

## Abstract

The literature reports that student transition between preclinical and clinical dental education can be traumatic and stressful for many reasons. Early clinical experience has been reported to provide some relief. In this qualitative study, twelve final year dental students were interviewed about their perceptions and experiences with a mentee/mentor (FOAL) program in Aarhus, Denmark, to see if it (1) counteracted stress perceptions from preclinical education to the clinic, (2) inspired professionalism and a sense of study relevance, (3) helped in learning to reflect on competencies and attitudes, (4) helped with clinical social perspectives (communication/contact), (5) helped with motivation to learn and (6) helped to reaffirm one’s professional study choice. Using qualitative description methods with purposeful sampling, data from interviews were collected, transcribed, analyzed and validated with a short questionnaire. The FOAL program, today, has several benefits for mentees, including partially helping in the preclinic to clinic transition and the increased insight into mentors’ clinical tasks and communication with patients. Informants described that FOAL also contributed positively to both mentee and mentor students’ learning motivation, collaborative skills and professional attitudes. Challenges were lack of organization/planning, not enough clinical hours, lack of clinical knowledge and persistent stress levels at the clinical transition. These issues are already being considered in the curriculum reform currently in progress and are also relevant to other dental curricula internationally.

## 1. Introduction

The literature [1,2,3,4] shows that the preclinic to clinic transition in dental education can be emotionally traumatic and stressful, which can lead to poor learning experiences [2], decreased motivation for learning [4] and, in the worst case, signs of anxiety and depression [1]. Building and maintaining motivation seems to be essential to averting poor outcomes [5,6,7]. Motivation for clinical learning has been applicably described in self-determination theory (SDT) [8,9,10,11], where degrees of motivation are divided into three main categories: amotivation, external motivation and internal motivation. SDT also clarifies that the quality of learning is based on the quality of motivation. Amotivation describes students with low or no motivation. In external motivation, students are affected by various external parameters that span several phases of development towards higher-quality motivation. The first and least autonomous phase is *externally regulated motivation*, where students study, for example, mainly because teachers or instructors have expectations of them and not because they themselves are optimally interested or engaged in learning or see its relevance or relatedness to becoming a professional. The next two phases of learning motivation are gradual transitions in a student’s improving self-motivation levels. The fourth phase of external motivation is *integrated regulation*, where students transition to becoming self-motivated. Finally, internal motivation is the most autonomy-building motivation due to engagement by students’ own volition, their needs for growth in self-awareness and social awareness and their sense of relatedness to the profession that they have chosen [9,10]. This means that in this phase, students are highly self-motivated to learn, wanting to show they have fully understood the relevance of the curriculum. As suggested above, SDT posits that these phases of motivation often progress from external to internal, depending on the student’s experiences during the study period [9,10]. Mentorship support is thought to specifically direct mentees towards creating an understanding of the value of their chosen profession and towards a gradual internalization of this value [11,12], which is a topic of interest in this paper.

Knowledge of early clinical experience as a teaching strategy and the theory of the benefits of these strategies are plentiful in the health literature [13,14,15,16,17]. As alluded to above, SDT proposes that different qualities of motivation affect different outcomes. There appears to be a link between early clinical experience and less stress [15,16] as well as the idea that students feel more confident about their future professional role at the clinic after these early experiences [14,17,18]. Dental students with early clinical experience have also been reported to have greater internal motivation for learning [5,6,7]. Since internal motivation favors optimal learning, the need for early clinical experiences for dental students seems to be supported by SDT [6,7]. As reasons for engaging in activities become more self-determined, positive outcomes are expected such as deeper learning, less superficial information processing, better performance, less intention to drop out, greater creativity and improved well-being or adjustment [11,16,19]. Mentorship support during the early years of medical education also appears to stimulate feelings of volition and needs for professional autonomy and relatedness, which are the tenants of SDT’s concept of internal motivation [11]. However, this is still a hypothesis in relation to dental education [6,7].

The present study aims to contribute to the international dental education literature and to inform educational policy considerations about early clinical experience and mentorship. It specifically evaluates student perceptions and experiences with a program at Fællesklinikken (FK), a comprehensive care clinic at the dental school in Aarhus, Denmark. To achieve this, specific aims were formulated. These took the form of the following research questions about early clinical experience as mentors and mentees and their implicit hypotheses:

**Hypothesis** **1** **(H1).** *Does early clinical experience help reduce stress/anxiety during the transition from preclinical education to the clinic?*

**Hypothesis** **2** **(H2).** *Is it inspiring to see older students treat patients (enhanced relevance to profession, according to SDT)?*

**Hypothesis** **3** **(H3).** *Did you learn to reflect on your own competencies and attitudes (enhanced self-awareness, according to SDT)?*

**Hypothesis** **4** **(H4).** *Did you learn about social perspectives (communication/contact) in the clinic (enhanced social awareness, according to SDT)?*

**Hypothesis** **5** **(H5).** *Did early clinical experiences increase your motivation for study activity and general learning within the education?*

**Hypothesis** **6** **(H6).** *Did you ever think of dropping out of dentistry? Did early clinical experience help you reaffirm your choice?*

## 2. Materials and Methods

### 2.1. Setting

The so-called “FOAL program” was first introduced, quite intuitively, to FK at the Aarhus Dental School. Here, first and second year students could acquire some experience at the clinic as respite from their heavy theoretical teaching load in the basic sciences. The program was formally introduced in 2012 in the form that it is now in in 2021, but there was also a program from 1979 to 2011, which was on a voluntary basis. That voluntary program was established at the time in an attempt to reduce first year drop-out rates, i.e., intended as a source of professional inspiration and motivation. Currently, dental students are required to be mentees 12 h per semester during the 1st–4th semesters.

The current mandatory FOAL program still occurs in the 1st–4th semesters, and FOAL students (mentees) act as assistants mainly for 7th–10th semester students (mentors) who have their own patients, since 5th–6th semester students work mostly in dyad pairs. Otherwise, in the first 4 semesters, students only have preclinical dental skill instruction in simulation clinics. Student contact with patients can only occur within the FOAL program. The clinical leader gives a lecture to 1st semester students as a short introduction to the teaching clinic and the role of foals. Mentee tasks under the program include suction assistance, entering measurement results in electronic journals and help in fetching materials, and, in some cases, a foal (mentee) may be allowed to polish off some work after depuration, or to carry out some other easier treatment tasks. Therefore, mentees get an introductory insight into the daily function of the teaching clinic before they themselves become clinicians. In addition, they also obtain opportunities to gain some social skills during the treatments, as in many cases, while sitting alone with patients, they have the chance to make independent contact.

### 2.2. Interviews

Twelve female final year Danish dental students aged 23–28 volunteered to be informants about their perceptions and experiences in the FOAL program using semi-structured interviews in Danish. Students were recruited in October–November 2020. Female dental students at the Aarhus School of Dentistry usually make up about 80% of the students, which is why only female participants were chosen in these interviews [20]. In this sample, 12 out of 46 possible female students were recruited. This was part of a purposeful sampling strategy [21] in which the women were recruited equally from each of the two clinical floors and with different clinical days of the week to cover any possible differences between student groupings and teacher influences. As they were in their final year, these informants had experience as both mentees and mentors, which was purposeful, since they could then reflect on both situations. Each interview was conducted, transcribed and validated among three female dental student co-authors. For anonymity, the subjects were code numbered from 1 to 12. These students were from the same class as subjects chosen for the study and were aware of many of the learning phenomena to be studied. They were given several hours of training in qualitative methods, interview techniques and transcription as instructed by the first author, who is both a clinical teacher and a senior researcher formally trained in qualitative methods. The questions are shown in Table 1.

To encourage more detail and greater in-depth answers, there were follow-up questions such as “Can you tell me more about that?” Thus, standard interview techniques were used that embraced qualitative description methods [22,23] such as confirmations and follow-ups, in order to check and supplement initial statements until each subject’s knowledge about topics appeared to be exhausted and the informant and interviewer agreed to stop. A quantitative validation method [24,25], in the form of a brief questionnaire, was also used in a follow-up re-visitation of the same informants. It consisted of sixteen true/false items based on group identification and the main findings of the students’ interview data. All 12 students subsequently completed the validation survey. The questions are listed in a table in the Results section.

The research was carried out in accordance with the Declaration of Helsinki and Aarhus Municipality’s ethical committee rules, including, but not limited to, that there was no potential harm to participants, that the anonymity of participants was guaranteed and that informed verbal and written consent of the participants was obtained.

### 2.3. Analyses

The students’ audio-recorded interviews were transcribed verbatim via the dictation program in Word 2016 and saved according to appropriate measures to ensure the anonymity of the subjects and their identities. The text files of the interviews were then imported into NVivo software [26] to assist with a general inductive analytic approach [27] to explore categories and compare contexts related to students’ experiences of the FOAL program. NVivo aids in structuring and analyzing the files, making it easier to find supportive material with concrete text quotations when exemplifying phenomena and common themes [27] which reoccur throughout the various interviews.

A “Code Book” [26] was prepared, in which brief descriptions with illustrative quotes for each category or theme developed in the NVivo program were documented. (The Code Book topics are listed in a table in the Results section.) To ensure that there was sufficient theoretical saturation, the data analysis ended only when there was redundancy of descriptions and no new categories of phenomena or themes could be found [27,28].

All student interviews and validation questionnaires were analyzed within their social contexts and were further defined by the following scientific assumptions: (1) that informants were members of the same socio-cultural group (Danish dental students of the same class, age and gender), (2) that they answered questions independently of each other and (3) that the questions were about a specific domain (early clinical experiences in the teaching clinic) [29].

The true/false survey provided the estimated validity and consistency in informants’ use of descriptions and findings from the entire sample, that is, estimates of consensus for the interviewed informants. This also made it possible to evaluate if there were sufficient numbers of informants for valid results at the 95% confidence level. Questionnaire responses were analyzed according to cultural consensus theory, which is based on a formal mathematical model [29]. This theoretical approach makes it possible to judge how accurately the informants described the relevant social phenomena in their responses, as well as the strength of agreement between informants. Unicet 6.0 software [30] was used to calculate these consensus and informant competency parameters. Unicet 6.0 provides Cronbach’s alpha calculations, factor analysis and Bayesian probability estimates based on patterns of subject agreement. Standardized values for intersubject agreement and minimum sample size required for testing at the given confidence levels, which are provided in the Results section and guided assessment of students’ results [31].

## 3. Results

The results in Table 2 below are the coded categories of student perceptions and descriptions of early clinical experience. Details of the coding descriptions and quotes in the text after the table correspond to the order of findings summarized in Table 2. As expected, students described a broad range of categories due to their “insider” role as users of early clinical experience. Results by specific aims and categories of findings are reported below, and many are accompanied by cogent quotations that illustrate each. Students’ quotations are identified as S1 to S12 for anonymity.

### 3.1. Overall Assessment of the FOAL Program

The informants mainly thought that the FOAL program had been positive, since as a mentee or foal, a student could acquire some early clinical experience and inspiration about their chosen profession. At the same time, there were also reports of shortcomings that had affected the experience. A good example of an overall assessment of the FOAL program is as follows:

S2: “It’s a good thing that in the first semesters they come up to get some experience at the clinic and that they are allowed to see and ask about things and get some knowledge about the clinic (and the profession). But they know nothing. So, it could be nice with some more introduction.”

Praise for the program in general, but also a lack of more introduction and theory, was also pointed out by another informant:

S5: “It was good to get up to the clinic early in the study and see how it works at the clinic and what and how to expect to work as a dentist. But it was also sometimes difficult to follow any theory behind what actually happened. So yes, you had seen a little, but still a little difficult to assess what had happened.”

### 3.2. Advantages with the FOAL Program

**As a mentee/foal**

** Students got to try something other than bookwork:** Informants lauded the FOAL program as an opportunity to try out their hands in the clinic rather than more basic sciences. Among other things, one informant formulated the following:

S4: “It was good to get a little away from the books, and come up to the clinic to see what you really will be doing someday.”

S3: “Then I was allowed to polish with a prophylaxis cup once, I can remember. It was mega cool!”

** Learning something about communication/contact with patients:** For better or worse, informants described different situations where they could appreciate the importance of good communication. As one informant put it:

S3: “You find out you like to have that kind of human contact, so you really have to talk with the patients. You have to make them feel comfortable and safe. I found that I actually think it is a cool way to work with people and the clinical environment up there.”

Another informant reported learning from watching mentors communicate with patients:

S6: “I actually think it was easier to see when there was not a very good communication between patient and mentor compared to when there was a good relationship and communication between them.”

Learning small talk was often part of the communication related to patient treatment, as described in the quote below:

S2: “There is a lot of small talk at the clinic. So it’s nice that you’re sometimes allowed to talk to patients a little bit before you (actually) have them yourself.”

** “Legitimized lack of knowledge”:** Informants described that as a foal, students do not have much experience or knowledge, neither about patients nor treatments, which is why it is not possible to hold them responsible for ignorance about theory or practice. Thus, many stated that it is better to just relax with expectations as a foal:

S5: “It was nice that they knew you were a foal. In this way you were allowed to ask more questions, i.e., legitimized ignorance. You didn’t really know enough. It was really minimal knowledge you had.”

S11: “I became less afraid of patient contact (with time), so it became more harmless. You can just sit on the sidelines quietly and learn things one step at a time. First you suction and then you can just sit and chat, since you are not in charge. Then you build up your knowledge up from there. I think that was really very good.”

** Opportunity to reduce the preclinical to clinical transition crisis in the fifth semester:** Theoretical learning during the first 2 years of dental education is supplemented with preclinical lab or simulation work, i.e., in the form of modules within the main clinical disciplines (restorative, periodontal, prosthetics, etc.). Learning these lab or simulation skills is part of the preparation to transition into the clinic environment. The FOAL program could also contribute to a less stressful transition according to many informants:

S11: “If you have not been a foal and you are in the fifth semester and have never sat in front of a patient before, it would be something completely different to have to go up and do your first filling or anesthesia. The FOAL program has contributed by making students familiar with the clinic and routines. They may even become familiar with the teachers. I definitely think it helps.”

Other informants stated that the FOAL program did not significantly reduce this stressful transition.

S5: “It makes the transition a little smaller. But, it is a very big leap to go from meeting up for 3 h at a time, sitting and suctioning, not really knowing what exactly has gone on and then to having your own patients. You have to call up patients and prepare for the whole treatment, with all that it entails. So yes, a big leap, still.”

** Experienced students are seen as role models:** Informants described that a foal records in their memory when their mentors are acting professionally, both in terms of behavior and treatment. The following quotations illustrate this.

S1: “You see how other older students handle situations and that they can be exposed to pressured situations. Yes, you have seen them talk to the patient and seen that they can make some small mistakes and thought yes okay, and that also worked out.”

In addition to seeing how mentors act with patients and the teachers, the foals also became familiar with the clinical environment from early participation.

S2: “There was a little everyday life at the clinic and you saw a little about what it was all about.”

**As a mentor**

** An extra pair of hands helps:** Overall, the informants as mentors thought that the great advantage of having a foal was the hands-on practical help they provide.

S4: “If you are in the middle of having trouble with something, you can ask: ‘Would you be so kind to fetch my teacher, because I need to stay with this in the meantime?”

** The mentee’s active engagement is crucial to the program:** According to the informants, the cooperation between a mentor and a foal depends on the level of interest and commitment of the foal.

S11: “It’s fun to have the younger ones up. Especially if it’s someone who wants to interact a lot and inquiries into things, not just suctioning a meter away. Then I think it’s fun to be a mentor.”

Informants added that interested and committed foals ask more questions, which is why the mentor, in addition to the practical help, also benefits from teaching them and testing their own theoretical knowledge.

S1: “You get to refresh your memory, because if they are curious and ask questions, you can explain what you are doing. Then you become even more confident (and say to yourself)—“Well, (I) actually know what (I am) doing!””

** Learning by teaching others:** As shown in the quote above, many informants explained that they learned by explaining treatments to foals, thus reaffirming their role as clinicians. In the process, mentors test their own knowledge by formulating and refreshing previously learned knowledge.

S3: “You do not always think about why you do this or that until there is someone sitting next to you and you have to explain it. Then you recall why you actually do things the way (you do) and more aware of your own way of operating.”

Simultaneously to the above, several mentors tried to make the treatment situation exciting and learned to be more educational. This pedagogical attitude was often inspired from the time they themselves were foals.

S2: “At least I try to make it exciting for them at the clinic, because I myself was a foal for a mentor who was not very inspiring to be with. So I teach and explain and allow (them) to get their fingers wet in the patient’s mouth. Just being allowed to touch a patient makes a big difference.”

As a mentor, you can also learn about your own stress reactions, and to relax during treatment, even when you have a new foal.

S6: “You want to have a good relationship with your patient, and if you have a relatively new foal, which you also have to start up, then you must at least learn (to) be able to relax with it all one way or another. Because it can quickly get stressful if it is also a new treatment that you are trying to learn.”

** No need for any special preparation to have a foal:** Most mentors did not prepare very much to have foal assistance. They did not think it necessary to prepare.

S4: “Many of the things we do come automatic, so I can explain without having to review a manual ahead of it.”

However, many mentors gave foals personal introductions before treatment began.

S11: “I always ask what semester they are in and if it is something they have done before. Then I tell them about the patient, and what we are going to do, and what we did last time. If it is a slightly special patient, who may present some challenges, then I also mention that.”

### 3.3. Challenges with the FOAL Program

** Told to fetch something, but the foal does not know what it is:** A significant challenge for foals at the clinic was described as their lack of knowledge about clinic organization, equipment, supplies and staff, since they were not introduced to this.

S6: “If you were sent out to find something, did not really hear what it was that the mentor said and you did not know who to ask for help, it could all be overwhelming.”

** Lack of knowledge about clinical dentistry:** Some informants remembered the feelings of being ignorant and unsure about how to behave as well as the requirements and expectations demanded of them.

S12: “I was very scared (at first) to do something wrong. I think it was enormously anxiety-provoking to have to sit with that suction and have no idea: “Does it hurt to get it on your tongue? Does it hurt to suction on the cheek? You sat and were completely panicky.””

**A scary social situation:** Coming up to the clinic as a foal and meeting patients, older students, teachers and staff can be very overwhelming for some new students. In some cases, some students experience it almost as anxiety-provoking, as expressed in this quote:

S7: “If you are actually a little unsure of yourself and you have to actively go in and ask ’I want to come and help you.’, it can be a personal challenge for a young foal; just to take that step and sit with strangers for 3 h.”

** “Lost, but cool”:** On the other hand, informants experienced both the challenges above as a foal and, at the same time, the exhilaration of being among clinicians in their chosen profession:

S10: “I was lost in a way because you do not really know people or the place (at first). You were just told to show up at a chair and you did. But I also felt a little buff, because we all knew that it was mega cool.”

** Lack of training of foals about the basics:** Although the informants described that they as mentors introduced their foals to treatments, there was a lack of basic mandatory training in being a foal, for example, to learn something about materials and equipment or the use of suction equipment.

S2: “I didn’t know the difference between a laser tip and a plastic instrument. You know nothing, so you should get a better introduction, since you feel a little stupid.”

** Focus of mentor’s attention to the foal can disappear with pressurized situations:** Besides lacking some type of basic training, the foals lacked orientation about what they could expect of mentors in stressful clinical situations:

S1: “If something is really difficult and I (as a mentor) am under pressure, then my focus of attention on the foal disappears. You have to have energy reserves in order to maintain attention to making explanations.”

**As a foal, you can feel more like a hindrance than a help:**

S2: “Sometimes the students you help are in pressured situations, so you feel more like a hindrance than a help. That’s how it is sometimes.”

** Competition for foal times at the clinic:** It could be urgent for either a foal or a mentor to find a partner because of periods with heavy demand. At the beginning of a semester, many foals want to help, which is why there may be competition between the foals to come up to the clinic and help. On the other hand, at the end of the semester, it is typically the mentors who find it difficult to get help, as foals have already fulfilled their foal times and are starting to study up for exams. These issues create stressful conditions for students.

### 3.4. Becoming Inspired as a Foal

Some informants said they were inspired by their foal experiences. Most found it inspiring that their mentors could be so proficient, and this had also motivated them to want to become proficient.

S3: “I just think it was really cool seeing how much they could do. I really wanted to be able to do the same. By being allowed to see someone good at their job, I also wanted to be good myself.”

### 3.5. Foals Reaffirming Their Choice of Profession

Most informants had considered and been in doubt about their educational choice to become a dentist. The most prominent reason for this was the stressful crisis that arose during the transition between their fourth and fifth semesters when starting at the teaching clinic. However, the FOAL program eased the difficult transition slightly. This is because, as a young student, you have an opportunity to gain insight into and engage in some clinical treatments, even though you are not a fully responsible clinical student yet.

S9: “Especially the fifth semester, I think, I was almost ready to drop it all. It was not so much FK, but more things around it that made it tiring. I liked FK. Do I think that the FOAL program helped to make me be less in doubt? Yes. Because there you just want to get started by seeing what they (mentors) sat and did, which made it quite clear. So, did I reaffirm my study choice as a foal? Yes, I think so.”

Another informant thought the FOAL program made little difference:

S12: “Yes, I have been in doubt many times, after we started at the clinic ourselves. There was all the pressure of seeing patients and at the same time having to juggle theoretical subjects on top of it. There were just too many balls in the air. So I think many times I thought “Oh, am I good enough for this, since now, again, I am sitting and fiddling around with an impression. The others can figure it out, why can’t I?”

This quote also indicates that self-doubt plays a prominent role in the preclinical to clinic transition crisis.

### 3.6. Considerations or Suggestions for Improvement

** Better distribution of times as foals:** The informants displayed a desire for a more structured FOAL program so that the planning schedule is optimized and there is no overlap between foal times and coursework.

S1: “One challenge is that it can be a little difficult if everyone from 1–3 semesters has coursework and (a mentor) needs a foal. Where there is small group coursework, that’s okay. But sometimes they all have a lecture that is at a really stupid time, like from 10 am to 12 noon, so you can’t get help then at all. So either they could put coursework either (earliest) in the morning or after clinic hours.”

S6: “(The school needs) to come up with something where different teams of foals are assigned certain days. Then it would not be as stressful to find someone when times are just totally booked.”

** Increase the number of mentee hours:** Many informants believed that more mentee hours will benefit clinical insight and reduce feelings of ignorance.

S10: “I think there could have been a few more hours because it can take a long time between times when you can be there. Then you lose a bit of (the routine). So maybe if there was one day a week where there was an hour or two where you could (flexibly) manage to be there, I think it would make more sense.”

** Not enough time for FOAL program in second year; best in first year:** Some informants pointed out that there is already a lot of pressure on students to complete theoretical courses in the second year, and that the FOAL program can be a stress factor. Thus, it would be better to complete all of the foal hours during the first year.

S9: “There may not have been enough foal hours in the first semester as it is right now. In the third and fourth semesters there is more pressure because you had several larger (theoretical) subjects.”

** Do simple treatments early as foals:** Several informants came up with the proposal to try small simple treatments on patients already in what is now the preclinical phase.

S12: “There are types of treatment you could perhaps start doing earlier (than the 5th sem). I think it could be cool because a simple periodontal treatment or instruction on tooth brushing or holding a mirror and probe at the same time is not something we’ve really done (systematically).”

Several informants also expressed a wish that foals would receive a “clinical mini-course” so that they were better equipped to start in the clinic.

** Have the same mentor every time:** Several informants as foals thought it best to have the same mentor every time they were at the clinic as it made them feel safe in the new and sometimes overwhelming setting. As mentors, they also agreed it was best to have the same mentee each time, as previously described above.

S5: “I think it was an advantage to be associated with a (specific) mentor or a group of mentors, to feel more secure. It gave more of a sense of the routine, since there was not just a new one every time.”

The results from the questionnaire responses are shown in Table 3 below. There was significantly high consensus and informant competence among the informants, suggesting, as shown in Table 4 below, that the sample size was adequate at the 0.95 confidence level, given the assumptions described in the methods.

## 4. Discussion

The purpose of this study was to investigate early clinical learning and evaluate the FOAL program’s influence on students’ perceptions at the Aarhus Dental School. Twelve last year clinical students’ perceptions and experiences about the benefits of and challenges with the FOAL program, as well as suggestions for improvements to the FOAL program, were explored.

There are important caveats to keep in mind regarding the present results. (1) Although these qualitative results may be representative of Danish female dental students, who are the large majority of Danish dental students, they may not be representative of males or other student nationalities. Cultural influences on student motivations and learning environments can vary from nation to nation and by gender. (2) No specific measures of stress or self-efficacy were used for comparisons with the results in this study. Efforts were focused solely on informants describing their own experiences and perceptions about early clinical experience as mentees and in mentorship in order to gain knowledge that would contribute to variable selection in future research and informed educational policy.

Overall, informants were positive about the FOAL program but often felt there was room for improvement. They described a large number of benefits, both as mentees and mentors. They also described that it was positive early in the curriculum to experience the clinical context of the profession and not just the theoretical studies that dominate the first two years. Informants described that the FOAL program helped to allay the crisis from preclinical education to the clinic, but only in part. Therefore, Hypothesis 1 could only be partially confirmed, but the program helped according to many informants. They had an opportunity to become familiar with the clinical environment, the teachers and the activities at the clinic. On the other hand, informants expressed that there should have been more time for foal activities since foals only received a limited insight into daily life at the clinic. Additionally, they stated that foals do not acquire an overall picture of the workload expected of clinical students, as they do not experience the extra tasks that come with being a responsible clinician, such as post-clinic lab work and making patient appointments. Many foals chose to use the same mentor because they felt that the learning environment was safer. On the other hand, this precluded opportunities to see different mentors’ work tasks, working methods and other ways of coping with the stresses of first year clinicians.

The present findings support a recent study by the Association for Dental Education in Europe (ADEE) about “Transition to Clinical Training in Dentistry” [4] which recognized early clinical experience as an important factor in achieving contextual learning and better integration of theory and practice [4]. These results support Hypothesis 2, that is, the FOAL program “provided professional inspiration and study relevance.”

There were also benefits on the mentor side of the mentor/mentee relationship. A student or resident, placed in the position of a teacher of near-peers, experiences a different relation to them [12]. Acting as a relative expert makes a mentor feel like an expert with feelings of competence, relative autonomy and self-esteem before others, which, in turn, can motivate the peer-teacher in further professional development [12]. Not only did mentors increase their self-awareness about their competencies (Hypotheses 3) but they also exhibited professional pride and attitudes when teaching young mentees.

Another advantage mentioned by several informants was that the FOAL program contributed to learning about contact with patients and communication, which supports Hypotheses 4. The ability to communicate clearly is an essential cornerstone skill for future professionals. Some informants reasoned that had it not been for the FOAL program, meeting patients and communication would have been an additional stress factor in the fifth semester, had they not tried it as a foal. This supports another recent English study that reported that dental student informants perceived early clinical experience to be an advantage in developing interpersonal skills for communication with patients and clinical staff, by acquiring gradual familiarity with the clinical environment [13].

As foals, many informants experienced increased internal motivation to learn more about theory as well as learning clinical skills (Hypothesis 5). Some even went so far as to say that had the FOAL program not existed, then their motivation would have suffered since it would depend solely on theoretical and laboratory teaching. A medical student study showed similar results in reports of acquisition of clinical knowledge and skills, self-confidence, improved performance, validation of career plans and increased motivation to study the basic sciences [14].

For most informants, the FOAL program contributed to reaffirming their choice of dentistry as a career (Hypothesis 6), since they had the opportunity to gain insight into clinical treatment, at the beginning of the education, and this makes it easier to confirm or deny the choice of dentistry as the correct career. Early clinical experience can lead to a full sense of volition, choice and self-determination [9,10]. This means that a student pursues an activity for the satisfaction that is derived from it regardless of external pressure [6]. Kusurkar et al. [19] also found that autonomous motivation in medical students correlated positively and significantly with not dropping out. On the other hand, the current mandatory 12 foal hours per semester may not provide sufficient experience to be able to make this decision.

Informants determined that one of the advantages for mentors, among others, was that an actively engaged foal could more likely test the mentor’s theoretical knowledge. This was perceived to give the mentor increased self-confidence and motivation by confirming their own mastery of clinical treatments and theory, i.e., professional identity, as described above. These findings support Hypotheses 3–5 since more actively involved foals contribute to greater learning benefits for both mentors and themselves. Thus, synergism in the mentor/mentee relationship promotes learning reflections, learning about communication and contact and learning motivation for both learners. Serrano et al. [4] described that self-motivation is regulated by satisfaction and performance. If a foal seems less involved, informants admitted that this did not contribute anything positive to a mentor’s learning curve. In another study, this lack of motivation (amotivation) was also found to be negatively correlated with medical students’ reflections about learning [19]. SDT concepts of controlled external motivation and amotivation are associated with negative outcomes, including low competence, poor well-being and insufficient psychological adaptation to university life [6].

The concept of self-efficacy in the context of mentee/mentor learning [32,33] also seems highly relevant to the discussion of the present results. Self-efficacy is a student’s “sense of being able to do” a task and/or “feeling confident or competent” to execute learned skills or attitudes in their education [34]. Self-efficacy is based on Bandura’s social learning theory [35] which posits that besides theoretical and directly conditioned learning, people also learn via (1) observation, (2) imitation and (3) modeling. Observational learning occurs constantly, and exposure to a mentor’s behaviors provides new learning through the modeling process. Studies on medical students have also confirmed that mentee/mentor relationships provided learning through observation, feedback and cognitive support [12,33,36], often even more than teacher contacts in some cases [37]. The present mentees had the opportunity to observe and consult with their mentors constantly, thus providing abundant opportunities for them both to build up self-efficacy and self-confidence. Thus, social learning theory is very fundamental to self-determination theory and professional motivational development [19]. Foals experienced, first-hand, multiple levels of learning by observing mentors with their patients. These are likely the first steps young dental students make in becoming more mature, acclimatizing to professional settings and identifying with their future role [17].

The interviews also revealed a number of challenges and needs for improvement in the current FOAL program. Almost all informants pointed out a lack of clinical knowledge as being their biggest challenge. This contributes to insecurity and can be overwhelming, since there are no experiences to fall back on. As a mentor, the clinical naiveté of the foal can also be a challenge if the mentor is already in a high-pressure treatment situation. The clinically naive foal may amplify the mentor’s irritation or frustration. Lack of knowledge about standards of treatment in a clinical situation can be frustrating or even a source of stress, according to Moore et al. [2]. The informants described that this problem was greatest in the first and second semesters and suggested a more structured orientation at the beginning of the FOAL program.

The current FOAL program revealed rifts about foal times as well as issues in relation to foals’ academic schedule. Therefore, another suggestion was to improve the distribution of times and thus provide a more structured FOAL program. Presently, the responsibility for achieving compulsory foal hours is placed on the individual student, which can contribute to increased stress and anxiety. Besides the obvious challenge of booking their own times, it also requires that a foal must confront social barriers with which he/she may not be comfortable. Thus, to confront these challenges, the informants suggested that a foal should be able to choose to work with the same mentor for all the hours, in order to provide security and better conditions for motivation and learning.

Another suggestion was that an even earlier start of clinical treatments should be implemented in the clinical curriculum. Earlier starts would have younger clinical students start with easier treatments on patients and would presumably provide additional experience, motivation and self-confidence to try gradually more difficult treatments. However, Dyrbye et al. [14] pointed out that even if one sees positive results with early clinical experience, more insight is still needed into how these affect students’ professional development. Perhaps an earlier start to clinical treatments in the third and fourth semesters, along with streamlined and clinically relevant theoretical support, as is now in the planning phase with a new curriculum reform at Aarhus, will ultimately provide an additional positive intrinsic motivation for students’ professional development. The idea seems at least good, in relation to trying to break the stress curve from the fourth to fifth semesters of the preclinical to clinic transition, which still exists at present (2021). At any rate, it seems that an improved FOAL program in the first and second semesters still has a lot of relevance and would provide a more nuanced and formative professional orientation for both foals and mentors.

## 5. Conclusions

The informants in this study felt that there were many benefits to the FOAL mentee/mentor program. They described, as mentees, that it helped them gain insight into the clinical world and the relevance of their studies, which contributed to a less stressful transition from the preclinical to clinical semesters. The study also found that a well-functioning FOAL program required an actively involved mentee, as this positively affected the mentee/mentor relationship and improved the motivation of both for learning, acquisition of social perspectives and professional reflection. In other words, mentees and mentors got out of the program what they put into it. Teachers in similar clinical learning situations need to point this out both in introductory orientations and in daily clinical experiences.

Although there is still pronounced stress among students in their transition to the clinic, the Aarhus Dental School is currently in the process of redesigning the curriculum to include an optimized early FOAL program in the first year combined with clinical transition to treating patients with easier treatments in the third and fourth semesters. The reflection on the present results indicates that this might provide an even greater benefit for the students that could further help eliminate the current clinical transition crisis period. These results should also have some interest internationally, since the Association of Dental Education in Europe has called for more attention to this problem.

## Figures and Tables

**Table 1:** Interview questionnaire items.

(1) What is your overall assessment of the FOAL program?
(2) What are the benefits of the FOAL program at the clinic as a mentee? … mentor?
(3) What challenges have arisen in connection with the FOAL program as a mentee?
(4) Have you had a feeling that you were inspired by your time as a mentee, i.e., as a foal?
(5) Are there (other) things you became aware of about yourself as a mentee? … mentor?
(6) Have you ever been in doubt about whether you wanted to become a dentist?
Has the FOAL program had an influence on it?
(7) Do you have something you would like to add in relation to the FOAL program?

**Table 2:** Categories of informants’ interview answers. (See details in text sections below by corresponding section numbers).

**Categories of Interview Findings re. FOAL Program**
**Overall assessment of FOAL program** = positive; needs improvements (Section 3.1)
**Advantages of the FOAL program** (Section 3.2)
**As mentees (foals):**
To try something other than bookwork
Learning something about communication/contact with patients
Legitimized lack of knowledge
Opportunity to reduce the preclinical to clinic transition crisis (5th sem.)
Experienced students are seen as role models
**As mentors:**
An extra pair of hands helps
Mentee’s active engagement is crucial to the program
Learning by teaching others
No need for special preparations to have a foal
**Challenges with the FOAL program** (Section 3.3)
Fetch something for a mentor, but you do not know what it is
Lack of knowledge about clinical dentistry
A scary social situation
“Lost, but cool”—feeling overwhelmed, yet exhilarated
Lack of training of foals about the basics
Focus of mentor’s attention to the foal can disappear with pressurized situations
As a foal, you can feel more like a hindrance than a help
Competition for foal times at the clinic
**Becoming inspired as a foal** (Section 3.4)
“Of course the older students were cool! I want to be too.”
**Foals reaffirming their choice of profession** It helped to be a foal (Section 3.5)
**Considerations or suggestions for improvement** (Section 3.6)
Better distribution of times as foals
Increase the number of mentee hours
Not enough time for FOAL program in 2nd year; best in 1st year
Do simple treatments early
Have the same mentor every time

**Table 3:** Questionnaire results: percentages of true vs. false answers for the 12 senior students.

Statistics: Consensus α Reliability = 0.94; Mean Competence = 0.74, SD = 0.20	T	F
(1) I am a female dental student in my 9th–10th semester.	100%	0
(2) I am between 20 between 30 years old.	100%	0
(3) I speak fluent Danish.	100%	0
(4) As a foal, there were no opportunities to see what the clinic was like, as well as what the training would lead to in the end.	33%	67%
(5) The first hours as a mentee in the clinic were overwhelming for many students for several reasons.	67%	33%
(6) As a foal, it was possible to experience that you belonged in “the clinical life”, even if you were not an official clinician yet.	83%	17%
(7) It was not an advantage if a foal was given the opportunity to do a simple treatment such as toothbrushing, rubber cup polishing or applying fluorides.	17%	83%
(8) A foal got the most out of mentors who could not maintain contact with them, even during pressured clinical situations.	0	100%
(9) As a foal, you had an opportunity to take part in a completely new social network, which was important for when you yourself would start up as a clinician.	58%	42%
(10) Both as a mentor or foal, there was no benefit in having the same partner from time to time, if it went well.	0	100%
(11) The FOAL program can help the often-stressful transition from pre-clinic to clinic by knowing a little about the clinic and the people there.	75%	25%
(12) As a foal, it did not help that mentors could put themselves in your shoes (empathy).	0	100%
(13) Mentors got the most out of active, curious foals, which affected the amount of explanation and teaching.	75%	25%
(14) As a mentor, you learn from teaching, i.e., meaningful communication reinforces what you have learned as well as your professional pride.	92%	8%
(15) The current FOAL program seems a bit disorganized, as not enough time was set aside for foal orientation or mentor training.	75%	25%
(16) Changing the number of foal hours and/or better distribution of hours would be advantageous.	92%	8%

**Table 4:** Standardized chart for minimal number of informants needed to classify a desired percentage of questions with a specified confidence level for various levels of cultural competence (note for clarity: 0.50 is considered “good” mean intersubject agreement according to [31]).

Proportion of Questions					
Answered “correctly”	Consensus (average level of cultural competency:)				
	0.5	0.6	0.7	0.8	0.9
At 0.95 confidence level:	(Minimal number of informants needed:)				
0.80	9	7	4	4	4
0.85	11	7	4	4	4
0.90	13	9	6	4	4
0.95	17	11	6	6	4
0.99	29	19	10	8	4
At 0.99 confidence level:					
0.80	15	10	5	4	4
0.85	15	10	7	5	4
0.90	21	12	7	5	4
0.95	23	14	9	7	4
0.99	>30	20	13	8	6

## Data Availability

The original data supporting the reported results are in Danish and can be requisitioned from the first author.
